# Association of Inflammatory Markers/Cytokines with Cardiovascular Risk Manifestation in Patients with Endometriosis

**DOI:** 10.1155/2021/3425560

**Published:** 2021-10-31

**Authors:** Uzma Rafi, Shaaf Ahmad, Syeda Shazia Bokhari, Muhammad Amir Iqbal, Amna Zia, Muhammad Amjad Khan, Nabila Roohi

**Affiliations:** ^1^Institute of Zoology, Physiology/Endocrinology Laboratory, University of the Punjab, Lahore, Pakistan; ^2^Department of Biology, Lahore Garrison University, Pakistan; ^3^King Edward Medical University, 54000 Lahore, Pakistan; ^4^Lady Willingdon Hospital, Lahore, Pakistan

## Abstract

This study is aimed at determining the association of inflammatory markers and proinflammatory cytokines with cardiovascular risk manifestation in women with endometriosis as compared to healthy controls. A total of 181 females of reproductive age with the absence of other inflammatory or autoimmune disorders and a lack of hormonal therapy for at least 6 months voluntarily participated in this investigation. Patients were 81 females, laparoscopically diagnosed with endometriosis, while the control group comprised 80 healthy females without any pelvic pathology. All subjects were 20-40 years of age. Exclusion criteria were diabetes, obesity, hypertension, metabolic diseases, cardiovascular, and renal disorders. C-reactive protein, fibrinogen, homocysteine, interleukin-17, and interleukin-33 were analyzed using commercially available ELISA kits. For statistical interpretation, the unpaired Student “*t*” test was used. All inflammatory markers and cytokines demonstrated elevated levels (*P* < 0.001) in endometriosis patients as compared to healthy controls. The results of the study revealed that the patients with endometriosis demonstrate a hypercoagulable status due to inflammation, which initiates atherosclerosis and associated complications. Hence, endometriosis can cause a risk of cardiovascular disorders in these patients.

## 1. Introduction

Endometriosis is a chronic, benign gynecological illness associated with infertility and pelvic pain. Despite having insufficient epidemiological data, it affects approximately 10% of women in their reproductive age [1]. Endometriosis seems to be asymptomatic in one-third of affected females but markedly found to reduce the wellbeing of infected individuals [2].

Endometriosis is involved in several immune-mediated events associated with the atherogenic lipid profile, heightened oxidative stress, and systemic inflammation [2, 3]. Various mechanisms are involved in the upregulation of inflammatory gene activation and dysregulation of hormone receptors. The elevated levels of these circulating markers for each of these processes have been observed in the serum as well as the peritoneal fluid of females with endometriosis compared with healthy subjects [4, 5]. It has been suggested that the markers of reactive oxygen species and oxidative stress are involved in several diseases like autoimmune disorders, chronic systematic comorbidities, and rheumatoid arthritis [6]. On account of a comparable process, it has been proposed that endometriosis could be associated with harmful effects on other body functions [7].

Knowing the association between endometriosis and systemic inflammation [8–10], it has been proposed that endometriosis may prompt these patients to a variety of disorders including multiple cancers, atherosclerosis, and inflammatory and endocrine disorders later in life [11].

Atherosclerosis [12] and endometriosis [13] both include tissue macrophages that articulate scavenger receptors and are exposed to lipoproteins. In endometriosis, both peritoneal fluid and atherosclerotic lesions are marked by the occurrence of growth factors, lipoproteins (LDL, HDL), oxidized LDL, macrophages, T cells, and cytokines derived from macrophages and T cells as well as chemotactic factors for T cells and macrophages [14].

An inflammatory condition, both in the peritoneal cavity and at the systemic level, can be observed in females with endometriosis. Peritoneal fluid, serum, and plasma of endometriosis patients demonstrated anomalous immune cells, as well as a differently expressed chemokine, prostaglandins, metalloproteinases [15], and cytokines due to inflammation, as compared to those of control subjects [16]. It has been reported that females with endometriosis revealed elevated levels of T and B lymphocytes in their peripheral circulation [17]. Moreover, elevated serum levels of tumor necrosis factor-alpha (TNF-*α*), interleukin-1 (IL-1), and interleukin-6 (IL-6) have been found, by some authors [18], in females with endometriosis. By considering the above-mentioned facts, it is prevalent that chronic inflammation may stimulate the process of atherosclerosis [19].

Numerous investigations demonstrated a higher rate of cardiovascular mortality and morbidity due to atherosclerosis in several patients with Crohn's colitis [20], systemic lupus erythematosus [21], rheumatoid arthritis [22], and Wegener's granulomatosis [23].

C-reactive protein (CRP), fibrinogen, and homocysteine are known markers of inflammation, and IL-17 and IL-33 are proinflammatory cytokines. C-reactive protein (CRP) is a primary indicator of tissue damage, infection, and inflammation in common clinical trials. CRP could be an additional potential biomarker in endometriosis [24]. Proinflammatory cytokines such as IL-1, IL-6, and TNF-alpha stimulate the synthesis of CRP [25] which are found to be overexpressed in women with endometriosis as compared to controls [8, 26]. In addition, increased CRP levels specify the inflammatory responses in patients, which may indicate the risk of developing colon cancer and metabolic and cardiovascular disorders [27]. Scarce data is available concerning the CRP level in the peripheral blood of endometriosis patients, which may be due to dissimilarity in patient selection, study design, and procedures applied to find out CRP levels [28].

Several prospective clinical trials have documented the association of elevated CRP with an increased risk of stroke [29], peripheral arterial disease [30, 31], myocardial infarction (MI) [32], and sudden death, both in men and women [30].

Fibrinogen, as a marker of inflammation and thrombosis, is found to be involved in cardiovascular disorders (CVD). Now it has been established that the increased fibrinogen level in plasma is remarkably involved with a thickness of intima-media and atherosclerosis [33]. Previous investigations have revealed the association between fibrinogen and ischemic stroke [34]. In the general population, fibrinogen has been proven to be a powerful and independent predictor of cardiovascular risk [35].

Due to the aggregation of platelets in endometriotic lesions, the females with endometriosis are apparently in a hyperfibrinolytic and hypercoagulable state [36]. In these females, platelet counts (PLT) and plasma fibrinogen levels increase [37] due to inflammatory processes which in turn initiate and promote coagulation, increasing the risk of microvascular thrombosis [38].

Hyperhomocysteinemia is a significant risk factor for inflammatory diseases including stroke, cancer, and cardiovascular disease [39]. Hyperhomocysteinemia is a result of inflammation, and this initiates oxidative stress which further stimulates inflammation [40]. Hence, inflammatory markers and increased homocysteine (Hcy) levels are commonly being observed simultaneously. Homocysteine and inflammatory markers can largely enhance the sensitivity of identification [41]. Moreover, elevated levels of the homocysteine in follicular fluid of females with endometriosis are also reported [42].

Interleukin- (IL-) 17 is a proinflammatory cytokine and plays a significant role in infection, mechanical injury, cancer, and inflammatory and autoimmune disorders [43]. This crucial cytokine is synthesized by T helper (T_H_17) cells and is known to play a detrimental role in the pathophysiology of endometriosis. Elevated concentrations of IL-17 and T_H_17 cells in peritoneal fluid as well as in serum have been reported in endometriosis patients as compared to endometriosis-free females and have also found to be involved in the disease progression [44]. IL-17 can also regulate macrophage induction through its receptors [45]. It is hypothesized that IL-17 may be a chemotactic cytokine and is associated with the pathogenesis of inflammatory disorders [46].

Interleukin- (IL-) 33 is a dominant member of the IL-1 family that has an influence on the innate and adaptive immune system and is significant in inflammatory disorders [47]. It is expressed in numerous cell types comprising epithelial and endothelial cells [48]. In response to mechanical stress, necrosis, and tissue damage, it is excreted into the extracellular matrix and predominately attaches to the ST2 receptor (suppressor of tumorigenicity 2) [49]. Myeloid and lymphoid cells of the immune system that include essentially mast cells, macrophages, natural killer (NK) cells, T cells, innate lymphoid cells, B cells, and neutrophils are stimulated by IL-33 via the ST2 pathway [50].

The peritoneal fluid and serum of endometriosis patients revealed elevated levels of IL-33 and sST2, compared to healthy women [51]. IL-33 plays a crucial role in the pathophysiology of various diseased conditions including fibrosis, inflammation, hypernociception, and vascularization, and all of these are also associated with the pathogenesis of endometriosis [52].

IL-17 and IL-33 are potential cytokines and key regulators of inflammatory responses and are reported to play a significant role in cardiovascular diseases such as dilated cardiomyopathy, myocardial ischemia, atherosclerosis, and myocarditis. Inflammation is considered the core of atherosclerotic cardiovascular disorders [53]. Remarkably, it mediates the atherosclerotic plaque's thrombogenic potential and plaque stability [54]. Oxidized low-density lipoprotein and hyperglycemia as well as hyperlipidemia stimulate the dysfunction of the endothelial cells via the expression of vascular cell-adhesion molecules [55]. The increased serum level of IL-33 and IL-17 is attributed to the myocardial damage [56, 57]. Elevated concentrations of IL-33 are threatening as it is released during necrotic cell death due to unchecked release of inflammatory cellular inclusions. It usually happens in tissue injury as a reaction to infection and inflammation [58].

The present study is aimed at finding out the association of inflammatory markers (C-reactive protein, fibrinogen, and homocysteine), proinflammatory cytokines (interleukin-17 and interleukin-33), and cardiovascular risk manifestation in females with endometriosis in comparison with the control. Our results will indicate the role of inflammatory and immunopathological mechanisms in disease progression and their detrimental effects on the cardiovascular physiology of female patients with endometriosis showing the significance of immune responses in the pathogenesis of cardiovascular disorders.

## 2. Material and Methods

This study involved a total of 161 women out of which 81 females were histopathologically and laparoscopically confirmed with endometriosis. Patients were staged according to the revised American Fertility Society (rAFS). All the 80 endometriosis patients studied had minimal/mild stage (stages I–II) and were in the luteal phase of the menstrual cycle.

For comparison, 80 females were matched for age and BMI, waist, and systolic and diastolic arterial pressure. These control subjects were without infertility or pelvic pain. The control group visited the hospital and underwent laparoscopy for different reasons like dysmenorrhea, ovarian cysts, and tubal ligation. The study was reviewed and permitted by the ethical review board of the regional tertiary care hospital. The study includes all of the control and diseased subjects fulfilling the inclusion criteria. Written approval from all the participants was received.

Control subjects constitute Group I and were not having any pelvic pathology confirmed by laparoscopy, whereas the females laparoscopically confirmed with endometriotic lesions comprise Group II. A comprehensive proforma was drafted to record the demographic variables of all subjects. Inclusion criteria for the selection of subjects in the study were as follows: the reproductive age of women, the absence of inflammatory disorders of the pelvic organs, chronic somatic pathology, and hormonal or drug medication for at least 6 months. Exclusion criteria include disorders including diabetes mellitus, arterial hypertension (arterial hypertension was defined as systolic blood pressure > 140 mmHg and diastolic blood pressure > 95 mmHg), renal or metabolic diseases, and obesity (BMI, ≥30 kg/m^2^). Similarly, subjects with a history of intrauterine fetal growth restriction, preeclampsia, and ovarian cysts other than endometrioma were also ruled out.

Sterilized disposable syringes (BD, Becton, and Dickinson, Singapore) were used to draw 5 cc blood from each of the subjects. The blood sample was then placed in a plasma vacutainer and was labeled with specific IDs of the subjects. Each blood tube was marked with the date and time of phlebotomy.

Within 3 hours of phlebotomy, separation of plasma was performed. Centrifugation was carried out at 3500 rpm for 10 minutes. Separated plasma was put into RNase-free, 1.5 mL microfuge tubes (Ambion, USA) and then stored at -80°C till further analysis.

Inflammatory markers including CRP, homocysteine, and fibrinogen (cat # PRS-00458hu, cat # PRS-01769hu, and cat # PRS-00616hu, respectively) and serum cytokines IL-17 and IL-33 (cat # PRS-00856hu and cat# PRS-00868hu) were analyzed with commercially available enzyme-linked immunosorbent assay (ELISA) kits of Glory Sciences Co., Ltd., USA. The Mindray MR 96a ELISA microplate reader was used for inflammatory marker assessment at the Physiology/Endocrinology Lab, Institute of Zoology, University of the Punjab, Lahore, Pakistan.

Data collected through biochemical analysis was statistically analyzed utilizing GraphPad Prism version 6. Unpaired Student “*t*” test was applied to compare the groups.

## 3. Results

**Figure 1:**
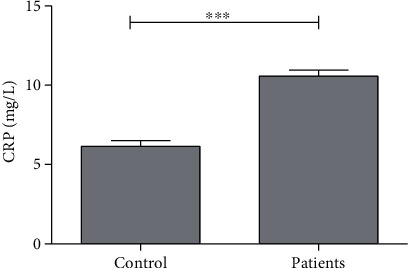
Average concentration of C-reactive protein (CRP) in control subjects and patients. ∗∗∗ indicates significance at *P* < 0.001.

CRP level was estimated to be 6.14 ± 0.35 mg/L in the control group, which elevated significantly (*P* < 0.001) by 72% in patients, whereas the level of CRP in the endometriosis group was 10.56 ± 0.39 mg/L (Table 1, Figure 1).

**Figure 2:**
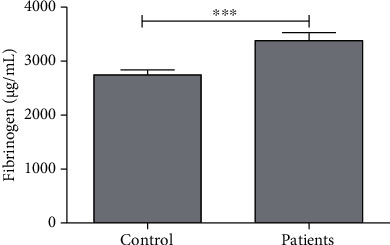
Average concentration of fibrinogen in control subjects and patients. ∗∗∗ indicates significance at *P* < 0.001.

The average level of fibrinogen was found to be 2749 ± 89.92 *μ*g/mL and 3382 ± 147.0 *μ*g/mL in the control and patient groups, respectively. A significant increase was observed with a 23% increase of fibrinogen in the patient group (Table 1, Figure 2).

**Figure 3:**
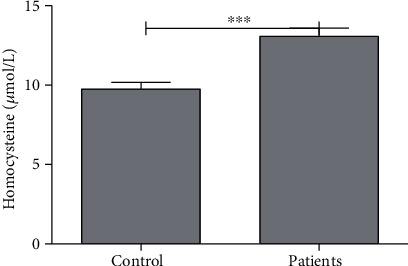
Average concentration of homocysteine (Hcy) in control subjects and patients. ∗∗∗ indicates significance at *P* < 0.001.

The average level of homocysteine in the control and endometriosis groups was found to be 9.76 ± 0.42 *μ*mol/L and 13.07 ± 0.52 *μ*mol/L, respectively. A significant difference (*P* < 0.001) was observed with a 34% increase of homocysteine in endometriosis patients (Table 1, Figure 3).

**Figure 4:**
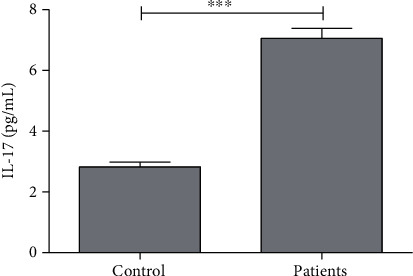
Average concentration of interleukin-17 (IL-17) in control and patients. ∗∗∗ indicates significance at *P* < 0.001.

IL-17 level in serum was observed to be 2.82 ± 0.15 pg/mL in healthy control which increased significantly (*P* < 0.001) by 150% in patients. The IL-17 level in endometriosis patients was 7.06 ± 0.32 pg/mL (Table 1, Figure 4).

**Figure 5:**
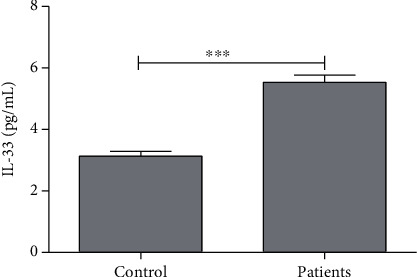
Average concentration of interleukin-33 (IL-33) in control and patients. ∗∗∗ indicates significance at *P* < 0.001.

The average level of IL-33 in the control and patient groups was found to be 3.12 ± 0.15 pg/mL and 5.52 ± 0.23 pg/mL, respectively. A significant difference (*P* < 0.001) was observed with a 77% elevation of IL-33, in patients with endometriosis (Table 1, Figure 5).

## 4. Discussion

In this study, women with endometriosis demonstrated a hypercoagulable and inflammatory state, manifested by elevated levels of CRP, homocysteine, and fibrinogen as well as IL-17 and IL-33. These are the markers of inflammation and are found to be associated with cardiovascular disorders.

CRP is not only a predictor but also a contributor, associated with the pathogenesis of CVD. It stimulates the growth of atherosclerotic lesions [59]. Higher levels of CRP also are involved in the formation of monocyte chemotactic protein 1 [60]. Furthermore, previous findings have demonstrated the adhesion molecules' expression in endothelial cells of humans [61]. Elevated CRP levels have been found to affect the activity of endothelial nitric oxide synthase and resulted in endothelial cell dysfunction [62]. The manifestation of atherosclerotic disorders is associated with monocytes/macrophages, endothelial cells, and vascular smooth muscle cells [63].

Still, there is a discussion concerning the CRP source, as it may be disputed that stimulated endothelial cells synthesize CRP, whereas smooth muscle cells of the human coronary artery can also produce CRP in response to stimulation by inflammatory cytokines [64]. Smooth muscle cell migration and proliferation is the significant incidence in atherosclerotic disorders [65]. In response to a proinflammatory mechanism, CRP can stimulate the functioning of NADPH oxidase 4 and Fc*γ*RIIa, thus promoting vascular smooth muscle cells to generate reactive oxygen species [66].

It has been demonstrated that in atherosclerotic lesions, CRP accumulation takes place earlier than that of monocyte infiltration, reflecting that CRP may impart a significant role in monocyte production [67]. CRP existence and complement activation in atherosclerotic plaques have been established in humans [68]. A group of researchers revealed the occurrence of complement proteins in human atherosclerotic plaques [67].

The endometriosis group in our study showed a significant increase in CRP levels as compared to the control, indicating increased CVD risk in these patients. Further investigation is required as to whether the CRP in atherosclerotic plaques specifies its involvement in the progression of atherosclerotic plaques and activates atherosclerosis by stimulating the complement system.

Fibrinogen is another marker of inflammation and is significantly elevated in females with endometriosis. It regulates platelet aggregation, blood viscosity, rheology, and thrombin formation. Elevated fibrinogen levels are reported in several diseases like nephrotic disorders and diabetes and are also associated with cardiovascular disorders [69]. Fibrinogen is strongly associated with hypercoagulation [70].

Several mechanisms are involved in endothelial dysfunction mediated by fibrinogen and its associated metabolites [71]. An ample amount of fibrin is associated with several atherosclerotic lesions which may be either deposited as wall thrombus in the plaque's intact surface or diffusely scattered all over the plaque.

Decreasing plasminogen concentration and fibrinolytic activity is associated with elevated fibrinogen and is also reported in cardiac atherosclerotic disease [72]. It has been observed that fibrin (intima) activates cell proliferation and cell migration and binds with fibronectin, which is further involved in adhesion and cell migration [73]. Fibrinogen and its metabolites present in the inner layer can enhance the permeability and vascular tone, collagen synthesis, and mitogenesis and also attract the leukocytes. In progressive atherosclerotic plaques, fibrin is also involved in the coupling of low-density lipoprotein (LDL) and lipid aggregation, leading to the formation of the lipid nucleus of atherosclerotic lesions [74].

Fibrinogen involves in the development of atherosclerotic plaque in the initial phases of coronary artery disease (CAD); hence, fibrinogen levels are strongly associated with CAD manifestation. Cardiovascular risk due to the effect of fibrinogen levels was found to be higher in young individuals and was similar to the effect of familiar risk factors such as diabetes mellitus, smoking, and hypertension. Hence, fibrinogen is a well-known inflammatory marker that seems to be involved in the prognosis and pathophysiology of cardiovascular disease (CVD). Its occurrence promotes the development of atheromatous plaque, in combination with other thrombotic molecules, inflammatory substances, and the endothelium. It also promotes the development of acute coronary syndromes.

In the present study, we also observed the association of the homocysteine and endometriosis. Women with endometriosis presented hyperhomocysteinemia (HHcy), which has long been regarded as a predictor for the development of deep-vein thrombosis as well as cerebral, coronary, and peripheral vascular disease [75]. Increased levels of homocysteine play a detrimental role via stimulation of the inflammation pathway, for instance, oxidative stress, endothelial dysfunction, the reduction of nitric oxide bioavailability, and leukocyte adhesion [76]. These components are also involved in the development of CVD. The risk of hyperhomocysteinemia not only is restricted to heart disorders but also can be expanded to incorporate other inflammatory disorders such as inflammatory bowel disease, Alzheimer's dementia, pregnancy complications, osteoporotic fracture, and neural tube defects [77, 78].

Homocysteine via several pathways is involved in the development of cardiovascular disorders, as it adversely affects the smooth muscle cells and vascular endothelium, triggering the modifications in arterial structure and function. Thus, HHcy is an independent risk factor for atherosclerosis leading to cardiovascular disorders [79, 80]. Numerous findings revealed a strong association between the severity of atherosclerosis and elevated levels of homocysteine and increased mortality due to CVD [81]. CVD risk due to homocysteine involves several mechanisms such as decreased circulating HDL levels, vascular muscle cell proliferation, thrombogenesis, and activation of an autoimmune response [82]. Homocysteine also stimulates NF-*κ*B (nuclear factor-kappa B), which is involved in the transcription of many genes generating immune and inflammatory reactions to decrease anti-inflammatory cytokines and upregulate proinflammatory cytokines [83]. Homocysteine also induces oxidative stress by downregulating the endothelial antioxidant defense via endothelial cell dysfunction; hence, there is an elevated intracellular concentration of reactive oxygen species (ROS) [84]. The precise mechanism of Hcy transportation in the vascular endothelium is not known; however, aortic endothelial cells in a human bind and bring in L-Hcy through at least four of the recognized cysteine sodium-dependent transport systems, that is, XAG, L, ASC, and A. XAG, L, ASC, and xc systems import L-homocysteine [85]. Hcy has an effect on vessels by altering the permeability of endothelial cells and contraction of smooth muscle cells through the suppression of nitric oxide synthase in endothelial cells, which synthesizes nitric oxide [86, 87]. Homocysteine may be involved in cardiovascular disorder due to the excess synthesis of hydrogen sulfide.

The current study demonstrated a markedly elevated IL-17 mean serum level in the patients' group as compared to the healthy control. Significantly elevated IL-17 serum level in patients with endometriosis has been reported earlier by Rosu et al. [88].

IL-17 is involved in disease progression as its higher concentration promotes inflammation and angiogenic pathways [89]. IL-17 is a significant cytokine of Th17 cells, which stimulates the production of several other factors like monocyte chemotactic protein-1 (MCP-1), TNF-*α*, IL-6, IL-8, IL-1*β*, and prostaglandin E2. All of these factors stimulate granulocyte production and neutrophil chemotaxis [90].

Th17 cells are key mediators of numerous inflammatory and autoimmune diseases; some of these diseases are inflammatory bowel disease, systemic lupus, autoimmune myocarditis, rheumatoid arthritis, scleroderma, psoriasis, multiple sclerosis, and endometriosis [91]. Th17 cells affect adaptive and innate responses and keep the intestinal mucosal barrier, thus preventing the movement of pathogens into the circulation from breaking mucosa via the chemotaxis of macrophages and neutrophils.

Several cells are involved in the production of IL-17 such as dendritic cells, T cells, macrophages, and natural killers. Thus, IL-17 regulates many different types of cells of the immune system and influences the expression of several chemokines, metalloproteases, cytokines, and chemokine receptors [92].

In rheumatoid arthritis, the patients were treated with methotrexate and adalimumab, the drugs responsible to decrease the levels of IL-17 [93]. Such types of studies demonstrated the effect of immunosuppressive drugs on the IL-17 level in treated patients.

IL-17 triggers proinflammatory responses at the inflammation site [94]. Numerous investigations have reported the role of IL-17, particularly, in response to inflammation that eventually leads to atherosclerotic disorder [95]. IL-17 has a dual action, showing protective as well as proatherogenic effects [96]. Patients with endometriosis, in our study, demonstrated elevated levels of IL-17; similarly, patients with acute coronary syndrome and cardiovascular disorders also showed increased numbers of circulating Th17 cells and related cytokines, like IL-17 [97].

An investigation on patients with coronary artery disease and healthy controls without any cardiac pathology revealed that interferon-*γ* (IFN-*γ*) and IL-17 were synthesized concomitantly and behaved synergistically to bring out inflammatory responses in vascular smooth muscle cells [98]. Moreover, dyslipidemia also stimulate IL-17 synthesis following aortic endothelial cell activation in humans and further adhesion of monocytes in vitro [99].

IL-17 levels are significantly increased in acute myocardial infarction and are associated with platelet aggregation [100]. Hence, IL-17 may upregulate the platelet aggregation in patients with chronic artery disorder. In an investigation, the inhibition of IL-17 or its related receptors is significantly associated with decreased atherosclerotic lesions [101].

Platelet aggregation has been reported to play a significant role in cardiovascular disorders including coronary artery thrombosis [102], peripheral vascular disease, and stroke [103]. Platelets may play a detrimental role in the progression of the atherosclerotic process.

Our results demonstrated the significantly higher serum concentration of IL-33 in the endometriosis group as compared with the healthy control group. IL-33 in humans is a pivotal cytokine and stimulates tubulogenesis as well as the production of angiogenic and proinflammatory cytokines [104]. The elevated level of IL-33 in patients with endometriosis and its involvement in disease progression by promoting inflammation and angiogenesis have been reported [105].

Recently, it has been recognized that IL-33 is a member of the IL-1 family and seems to be expressed in several cells such as smooth muscle cells, epithelial cells, endothelial cells, keratinocytes, fibroblasts, activated macrophages, and dendritic cells [106]. IL-33 exhibits a heterodimer receptor comprising of accessory proteins, IL-1, and ST2 receptors. The ST2, via splicing, encode for 3 isoforms of ST2 proteins: a transmembrane receptor (ST2L), a variant form present in the human gut (ST2V), and secreted soluble ST2 form acting as a decoy receptor for IL-33 (sST2) [107].

The elevated serum level of IL-33 in the endometriosis patient group was attributed to myocardial damage and cardiovascular disorders. Studies on IL-33 have demonstrated that as IL-33 is mostly secreted during necrosis which takes place as a result of trauma, infection, or tissue damage, hence, IL-33 elevation can be regarded as an alarmin [108, 109]. Various investigations also revealed that IL-33 is a multifunctional cytokine showing anti- as well as proinflammatory properties as determined by the disease [110].

Endometriosis and cardiac disorders share mutual pathogenic mechanisms via immunologic and genetic susceptibility, hormonal deviations, and active endometrial tissue [7]. Various studies reported that the effect of IL-33 in cardiac patients became worse, as an increasing level of this cytokine prognosticates cardiovascular disease events.

Some previous findings regarding IL-33 concentration reported its useful effect on cardiovascular disorders such as cardiac fibrosis, atherosclerosis, hypertrophy, and diabetes [111]. It has been demonstrated that Th2 cells are involved in the expression of ST2L but not regulatory T cells or Th1 cells; hence, these Th2 cells stimulate IL-33 which is associated with the activation of Th2 cytokines such as IL-13, IL-5, and IL-4 cells [112]. It has been suggested that an imbalance of Th1/Th2 may be associated with the progression of ischemic heart diseases [113].

In this present investigation, we did not evaluate the sST2 concentration as an IL-33 decoy receptor. However, previous studies revealed that sST2 concentration elevated significantly in cardiovascular disorders including myocardial infarction, coronary artery disease, atherosclerosis, ischemic stroke, acute aortic dissection, and giant-cell arteritis [108, 114]. Hence, measurement of sST2 concentration can facilitate in better understanding of IL-33 contribution in cardiovascular disorders. In conclusion, our results showed that the higher serum concentrations of IL-33 were associated with ischemic heart disease [56].

## 5. Conclusions

Endometriosis is an inflammatory disorder associated with an increased risk for cardiovascular comorbidities. To treat these patients, a multidisciplinary approach is highly recommended. Further studies are required to resolve the issues associated with endometriosis and cardiovascular disorders such as endometriotic plaque thickness, dose-dependent risk of CVD, and endometriosis severity and the role of immunological and decoy receptors in disease progression. Immunological markers including IL-33 and IL-17 and their associated pathways are key players in establishing a link between endometriosis and CVD. Results of immunosuppressive drugs in clinical trials may further provide evidence and show the effectiveness of these therapies. It is essential to promote increasing awareness regarding endometriosis and CVD association. Further investigations and screening of endometriosis patients might help prevent or delay the manifestation of cardiovascular disorder and reduce the CVD burden.

Hence, inflammation plays an indispensable role in atherosclerosis and subsequent CVD. Various pathways have been recognized, but these may not impart to the development of CVD equally. Furthermore, diverse medical conditions may involve distinct mechanisms. The capability to precisely evaluate the mechanisms involved in CVD risk in different groups of patients depicts an actual question of recent research. Assessing which pathway or mechanism(s) are pivotal in patients will direct us when and how to intervene. Several drugs used in clinical practice such as monoclonal antibodies, which are specifically aimed at different targets involved in inflammatory interactions, may prove valuable tools towards the control of CVD.

Although markers of inflammation are used widely in clinical practice and several epidemiological studies have evaluated their role in CAD, there are still aspects that need further investigation. Thus, many more large-scale studies are required to evaluate the association of inflammatory markers with advanced stages of endometriosis and atherosclerosis.

## Figures and Tables

**Table 1:** Inflammatory markers/cytokines and demographic variables in comparable groups. Values are mean ± SEM.

Parameters	Control (*n* = 80)	Endo (*n* = 81)	*P* value	Percentage difference
Age (yrs)	31.01 ± 0.62	32.1 ± 0.58	0.2	3
SBP (mmHg)	122.6 ± 0.98	123.1 ± 0.98	0.7	0.4
DBP (mmHg)	80.63 ± 0.98	82.04 ± 0.90	0.2	2
Waist (cm)	84.8 ± 0.55	85.17 ± 0.76	0.6	0.4
BMI (kg/m^2^)	22.5 ± 0.28	23.26 ± 0.30	0.09	3
CRP (mg/L)	6.14 ± 0.35	10.56 ± 0.39	<0.001	72↑^∗∗∗^
Fibrinogen (*μ*g/mL)	2749 ± 89.92	3382 ± 147.0	0.003	23↑^∗∗∗^
Homocysteine (*μ*mol/L)	9.76 ± 0.42	13.07 ± 0.52	<0.001	34↑^∗∗∗^
IL-17 (pg/mL)	2.82 ± 0.15	7.06 ± 0.32	<0.001	150↑^∗∗∗^
IL-33 (pg/mL)	3.12 ± 0.15	5.52 ± 0.23	<0.001	77↑^∗∗∗^

∗∗∗Indicate significance at *P* < 0.0001. SBP: systolic blood pressure; DSP: diastolic blood pressure; BMI: body mass index; CRP: C-reactive protein; IL-17: interleukin-17; IL-33: interleukin-33.

## Data Availability

The datasets used during the current study are available from the corresponding author on reasonable request.
